# The impact of dementia on service use by individuals with a comorbid health condition: a comparison of two cross-sectional analyses conducted approximately 10 years apart

**DOI:** 10.1186/s12916-018-1105-8

**Published:** 2018-07-24

**Authors:** Holly Q. Bennett, Sam Norton, Frances Bunn, Louise Robinson, Greta Rait, Claire Goodman, Carol Brayne, Fiona E. Matthews

**Affiliations:** 1 Cambridge Institute of Public Health, Forvie Site, Robinson Way, Cambridge, CB2 0SR UK; 2 Institute of Psychiatry, Psychology & Neuroscience, 16 De Crespigny Park King’s College London, London, SE5 8AF UK; 3 Centre for Research in Public Health and Community Care, University of Hertfordshire, College Lane, Hatfield, Hertfordshire, AL10 9AB UK; 4 Newcastle University Institute for Ageing and Institute for Health & Society, Newcastle University, Newcastle Biomedical Research Building, Campus for Ageing and Vitality, Newcastle upon Tyne, NE4 5PL UK; 5 Research Department of Primary Care and Population Health, UCL Medical School (Royal Free Campus), Rowland Hill Street, London, NW3 2PF UK; 6 MRC Biostatistics Unit Cambridge Institute of Public Health, Forvie Site, Robinson Way, Cambridge Biomedical Campus, Cambridge, CB2 0SR UK

**Keywords:** Dementia, Co-morbidity, Care, Change over time

## Abstract

**Background:**

The presence of concomitant medical conditions in people with dementia is common. Dementia may be related to differential use of health, social and informal care.

**Methods:**

Data from two large UK population-based studies (CFAS I & II) of adults aged 65 years and older were analysed using logistic regression for binary outcomes and Poisson regression for count outcomes to look at differences in non-dementia service use by those with dementia and a health condition in comparison to those with the health condition alone.

**Results:**

A total of 1619 individuals from CFAS I and 3805 individuals from CFAS II over the age of 75 years were included in this analysis. The presence of dementia and either stroke, diabetes or visual impairment tended to increase the likelihood of a range of different services being used over having any of the health conditions alone. There has been a shift to the use of unpaid care over time. There is now increased use of unpaid care from friends and family by those with dementia and another health condition in comparison to the health condition alone.

**Conclusions:**

Either due to the decrease in care home spaces or affordability, people with dementia are now relying on unpaid care more than people with other long-term health conditions.

**Electronic supplementary material:**

The online version of this article (10.1186/s12916-018-1105-8) contains supplementary material, which is available to authorized users.

## Background

Due to population ageing and since its prevalence doubles every 5 years after the age of 65, dementia is a major public health concern [1]. The course of dementia is complex and variable, and it is associated with multi-morbidity, disability and frailty [2–5]. Dementia affects global functioning (e.g. ability to plan and organise self-care and care of others, to remain socially and economically active) and the needs of the person with dementia syndrome are often long term and cumulative [6].

People with dementia also have co-morbidities, requiring access to different health and social care resources. The prevalence of comorbid conditions in people with dementia is considerable and there is increasing interest in the impact of comorbidities on people diagnosed with dementia [7, 8]. There have been many changes to health and social care over the last two decades. As there are now less people moving into care settings in the UK the service use profile of those living in the community is likely to have changed, with potential increases in the use of health and social care services over time.

This analysis is part of the Comorbidity and Dementia Study, a mixed methods study that aimed to explore the impact of dementia on access and use of non-dementia services for those with dementia and a co-existing health condition [9]. The aim of this analysis was to compare service use by individuals with dementia living in the community at two time points, especially in light of the fact that less people are now moving into care settings.

## Method

### Sample

The sample was drawn from the Cognitive Function and Ageing Studies (CFAS I and CFAS II; see http://www.cfas.ac.uk). CFAS I and CFAS II are UK-based, multi-centre, longitudinal population studies using near identical designs with the primary aim to estimate the prevalence and incidence of dementia and cognitive impairment [10, 11]. Both CFAS I and CFAS II randomly sampled individuals aged 65 years or older from the Family Health Service Authority lists in Newcastle, Nottingham and Cambridgeshire. These three areas in England were selected to provide good geographical spread across urban and rural locations. Baseline interviews began in 1991 for CFAS I, with an 80% response rate [12], and in 2008 for CFAS II, with a 56% response rate [13].

Baseline interviews included questions about sociodemographic characteristics, lifestyle, health, activities of daily living and cognition. Questions about the presence of health conditions were asked at each interview time point. Questions on service use were introduced for the whole CFAS I sample in the 10-year follow-up interview. At this point, individuals in CFAS I were, by definition, aged 75 years and above, thus the CFAS II sample for this analysis was restricted to the same age range. Furthermore, to understand use of health, social and informal care in the community, individuals living in care homes were excluded (CFAS I *n* = 109, CFAS II *n* = 197). 

### Dementia

For both the CFAS I 10-year follow-up assessment and the CFAS II baseline assessment, dementia was diagnosed by an algorithmic approach using the Geriatric Mental State (GMS) Automated Geriatric Examination for Computer Assisted Taxonomy (AGECAT). This is based on the GMS interview. This standardised interview for ascertainment of dementia and other neuropsychiatric syndromes in the older population provides high agreement with the Diagnostic and Statistical Manual of Mental Disorders (DSM) IIIR criteria (kappa = 0.86 [14]) and, if all information was not available for the GMS-AGECAT, a diagnostician (physician) would provide the study diagnosis of dementia from DSM IIIR criteria using information from the participant and informant interviews as well as formal interviewer observations. Cognitive status was also assessed using the Mini Mental State Examination [15].

### Other health conditions

The impact of three exemplar ‘target’ conditions – diabetes, stroke and visual impairment – was considered to help determine the influence of co-morbid dementia on the use of non-dementia service. Also included were angina, Parkinson’s disease, hypertension, anaemia, breathing difficulties, transient ischaemic attack, heart attack and hearing difficulties. These conditions were chosen because they are common in older people, generally involve some form of external monitoring and require collaboration between primary and secondary care. Moreover, they may exacerbate or influence the progression of dementia and management of these conditions, and self-management in particular is likely to be complicated by the presence of dementia [7, 16]. All health conditions apart from cognition were self-reported (respondent and informant) in both CFAS I and CFAS II. Having a target condition was defined as having one or more of stroke, diabetes or visual impairment.

### Service use

All services, including health, social and informal care, were also self-reported by the respondent only. Informants were not asked about service use at the 10-year follow-up wave of CFAS I and therefore had to be left out in CFAS II for comparison. There were questions on services used day to day, service use in the 4 weeks prior to interview and hospital service use. Day-to-day services refer to any relevant services (including unpaid care) that help the participant with any activities of daily living. These included home help, a care worker, Meals on Wheels, a community worker, a community nurse, a warden, paid help, other professional services and unpaid care. Questions asked on service use in the previous 4 weeks included home help, any nursing service, a chiropodist, Meals on Wheels, a physiotherapist, an occupational therapist, a speech therapist, a social worker, day centres, day hospitals and a general practitioner (GP). Some day-to-day services were also included in services used in the 4 weeks prior to interview as they are services helping with activities of daily living but may not reflect every day need. Visiting the emergency department in the last 3 months, being a day patient in the last year and being an inpatient were included in hospital services.

### Statistical analysis

All estimates were inverse probability weighted to account for study design and non-response to the initial invitation to participate in the baseline interview. For the CFAS I analysis, weights were computed based on birth cohort, sex and deprivation status based on postcode. Additionally, since the CFAS I sample is based on the 10-year follow-up assessment, weights were also adjusted for attrition based on age, sex, stroke, diabetes, visual impairment and last known cognition status (Mini Mental State Examination). For CFAS II, weights were computed using age, sex and deprivation status.

Adjusted rates of service use were estimated using logistic regression for binary outcomes and zero-inflated Poisson regression for count outcomes. All models were adjusted for age and sex. The comparison was made between dementia with a health condition and the health condition by itself as the referent. Analyses could not be adjusted for deprivation as well as age and sex due to small group size. A sensitivity analysis was conducted to compare sex- and age-adjusted models to deprivation only-adjusted models. All analyses were undertaken using Stata version 12.1, and presented with 95% confidence intervals (CIs). An incidence rate ratio (IRR) or odds ratio (OR) estimate of ≤ 0.7 or ≥ 1.4 were used to determine potentially important clinical effects to highlight regardless of whether the 95% CI indicated statistical significance or not.

## Results

After excluding those not living in the community, 1619 remained in CFAS I and 7599 remained in CFAS II. To match individuals in CFAS I, only those aged 75 years or above were included from CFAS II, resulting in 3805 individuals being included in the CFAS II analysis. Table 1 gives the prevalence of the target health conditions for two time points in those aged 75 years or more. The prevalence of stroke was similar in both studies but the prevalence of both diabetes and visual impairment increased slightly between the CFAS I 10-year follow-up and CFAS II (Table 1). Table 2 gives the prevalence of each of the target health conditions by dementia status. The increases seen in diabetes and visual impairment were in those that did not have dementia (Table 2).

**Table 1:** Weighted prevalence of dementia and the target health conditions of those living in the community aged 75 and above for CFAS I and CFAS II. See methods for description of weights

Health condition	n	Weighted %	95% CI
CFAS I 10-year follow-up (*n* = 1619)			
Dementia	83	6.4	3.9–10.3
Stroke	160	9.7	7.6–12.4
Diabetes	152	8.1	6.3–10.4
Visual impairment	178	12.5	9.3–16.5
Target comorbidities	416	26.3	22.3–30.6
CFAS II baseline (*n* = 3805)			
Dementia	277	8.5	7.5–9.6
Stroke	377	10.4	9.4–11.4
Diabetes	540	14.5	13.3–15.7
Visual impairment	602	17.3	16.0–18.6
Target comorbidities	1294	34.5	33.0–36.1

**Table 2:** Weighted prevalence of the target comorbidities separated by dementia status for those aged 75 and above living in the community for CFAS I and CFAS II. See methods for description of weights

	Dementia			No dementia		
Health condition	n	Weighted %	95% CI	n	Weighted %	95% CI
CFAS I 10-year follow-up (*n* = 1619)						
Stroke	17	22.6	14.0–34.4	143	9.7	8.3–11.4
Diabetes	13	15.6	6.6–34.8	139	8.6	7.3–10.1
Visual impairment	19	21.7	13.5–32.9	159	11.4	9.7–13.2
Target comorbidities	41	47.6	36.5–58.8	375	25.4	23.2–27.7
CFAS II baseline (*n* = 3805)						
Stroke	47	17.9	13.3–23.8	330	9.7	8.8–10.8
Diabetes	42	16.2	11.8–21.9	498	14.3	13.2–15.6
Visual impairment	39	16.7	12.1–22.6	563	17.3	16.0–18.7
Target comorbidities	100	35.7	29.7–42.1	1194	35.1	33.5–36.8

### Dementia and stroke

The results comparing service use by individuals with dementia and stroke to those with stroke by itself for CFAS I and CFAS II are given in Additional file 1: Table S1. The number of visits to inpatient services was increased in CFAS I and CFAS II by those with dementia and stroke in comparison to stroke alone (Additional file 1: Table S1). In CFAS I, the number of services used day to day was increased (IRR 2.0, 95% CI 1.3–3.1) but this was not the case in CFAS II (Additional file 1: Table S1). This could be due to the increased use in home help (OR 2.8, 95% CI 0.4–18.0) and paid help (OR 1.4, 95% CI 0.3–7.5) in CFAS I, whereas in CFAS II, use of home help was not increased and use of paid help was decreased slightly by those with dementia and stroke compared to those with stroke alone (Additional file 1: Table S1). Day-to-day use of a care worker was similarly high between studies and use of unpaid care was increased in CFAS II but not CFAS I (Additional file 1: Table S1). There was increased use of nursing services (OR 2.2, 95% CI 0.9–5.2) and a chiropodist (OR 2.1, 95% CI 1.0–4.2) in CFAS II but not in CFAS I (Additional file 1: Table S1) and the use of home help and a day centre was increased in both studies (Additional file 1: Table S1). Generally, there was not much difference in the use of hospital services between studies, with use of inpatient services being increased in both studies (Additional file 1: Table S1).

### Dementia and diabetes

Additional file 1: Table S2 gives results for CFAS I and CFAS II comparing service use in individuals with dementia and diabetes to those with diabetes alone. Between studies, the number of services used by those with dementia and diabetes compared to those with diabetes alone was similar (Additional file 1: Table S2). Out of the individual day-to-day services, there was decreased use of paid help in CFAS II (OR 0.1, 95% CI 0.0–0.8) but not in CFAS I (Additional file 1: Table S2), and there was increased use of unpaid care in CFAS II (OR 2.8, 95% CI 1.3–5.9) but not in CFAS I (Additional file 1: Table S2). Those with dementia and diabetes had help from a day-to-day care worker more than those with diabetes alone in both studies (Additional file 1: Table S2). In the 4 weeks leading up to the interview, those with dementia and diabetes compared to those with diabetes alone used home help (OR 3.7, 95% CI 1.4–9.4) and nursing services (OR 2.4, 95% CI 1.1–5.4) more in CFAS II than in CFAS I (Additional file 1: Table S2). The use of a day centre was increased in both CFAS I and CFAS II (Additional file 1: Table S2). Hospital use between CFAS I and CFAS II was similar.

### Dementia and visual impairment

Results for CFAS I and CFAS II comparing service use in individuals with visual impairment and dementia to those with visual impairment without dementia are shown in Additional file 1: Table S3. The number of inpatient visits to hospital was increased for those with dementia and visual impairment compared to those with visual impairment alone in both studies (Additional file 1: Table S3). Day-to-day home help was increased in CFAS I (OR 2.1, 95% CI 0.2–19.4) but not in CFAS II (Additional file 1: Table S3). Unpaid care was increased in CFAS II (OR 2.9, 95% CI 1.3–6.7) but not in CFAS I (Additional file 1: Table S3). Use of a care worker day to day was similarly increased in both studies but use of paid help was decreased in both studies (Additional file 1: Table S3). The use of a chiropodist was increased in CFAS II (OR 1.8, 95% CI 0.9–3.8) but not in CFAS I (Additional file 1: Table S3). Use of home help in the previous 4 weeks before interview was increased at both time points (Additional file 1: Table S3). Casualty hospital services were increased in CFAS I (OR 1.9, 95% CI 0.4–8.5) but not in CFAS II and inpatient hospital service use was increased in both studies (Additional file 1: Table S3).

### Dementia and other health conditions

In general, similar use of services was seen in the other health conditions as well. Increased use of a care worker day to day in both CFAS I and CFAS II was apparent in those with dementia and all other health conditions apart from Parkinson’s disease, where the number of people with dementia and Parkinson’s disease who used the service were too low to complete analysis (Additional file 1: Table S4–S11). Unpaid care was increased in CFAS II and not CFAS I for angina (Additional file 1: Table S4), Parkinson’s disease (Additional file 1: Table S5), breathing difficulties (Additional file 1: Table S8), transient ischaemic attack (Additional file 1: Table S9), heart attack (Additional file 1: Table S10), and hearing difficulties (Additional file 1: Table S11), whereas for hypertension (Additional file 1: Table S6) and anaemia (Additional file 1: Table S7) unpaid care was increased in CFAS I and CFAS II. In most other health conditions where there were the numbers to complete analysis, day-to-day paid help was decreased in both studies, rather than just in CFAS II (Additional file 1: Table S4-S11). Home help use in the 4 weeks prior to interview was consistently increased in both studies for the other health conditions (Additional file 1: Table S4–S11). Day centre use in the 4 weeks prior to interview was also generally increased in both studies, where it was possible to complete the analysis. Increased nursing service use in CFAS II but not CFAS I was also seen in anaemia (Additional file 1: Table S7) but, otherwise, use was similar between studies in other health conditions. Increased use of inpatient hospital services in both studies was only seen in breathing difficulties (Additional file 1: Table S8) and transient ischaemic attack (Additional file 1: Table S9), otherwise it was increased in CFAS I but not CFAS II for angina (Additional file 1: Table S4), hypertension (Additional file 1: Table S6), heart attack (Additional file 1: Table S10) and hearing difficulties (Additional file 1: Table S11).

The sensitivity analysis conducted showed that most estimates from the deprivation adjusted models were within CIs for the age- and sex-adjusted models for CFAS I and CFAS II (Additional file 1: Table S13–S24). The only exception was the estimate comparing the number of day-to-day services accessed by those with angina and dementia in comparison with angina alone in CFAS I (Additional file 1: Table S16). This does not change any of the conclusions, as the estimate became stronger, from IRR 1.6 (95% CI 1.1–2.3, age and sex adjusted) to IRR 2.9 (95% CI 1.8–4.8, deprivation adjusted).

## Discussion

Dementia is common in the older population and many people living with dementia have concomitant medical conditions. Prevalence rates for dementia, stroke and visual impairment did not change substantially between the two studies amongst those over the age of 75; however, diabetes prevalence has increased. Service use among people over the age of 75 with dementia and the selected comorbidities considered in this paper has indicated that, over the last 10 years, some changes have been seen for those with dementia and a health condition. Unpaid care is now accessed more by those with dementia and a health condition than those with the health condition only, whereas this was mainly not the case before. Use of paid help, on the other hand, was decreased in CFAS II for those with dementia and another health condition and was also mainly decreased in CFAS I too, which could indicate lack of availability or access. Inpatient hospital services use was generally seen in CFAS I by individuals with dementia and a health condition, but this was not always the case in CFAS II.

The main strength of this piece of work is that these estimates have come from two large population-based, randomly sampled studies at different time points, which can be directly compared to each other. In addition, there was an advantage to using an algorithmic approach to diagnosis of dementia since it remains consistent across both studies, whereas the clinical diagnosis of dementia has changed. Although the original sample size was large, use of individual services and prevalence of some health conditions were low, resulting in wide CIs. Results can still indicate increased or decreased service use; here, IRR or OR estimates ≤ 0.7 or ≥ 1.4 were used to indicate clinical relevance rather than statistical significance, but should be interpreted with caution as, due to the number of variables (all defined a priori), some associations will have been expected to have occurred by chance alone.

One of the limitations was the cross-sectional design of the analysis – although CFAS I had longitudinal data, the service use questions were introduced at a late stage and attrition after this point meant that longitudinal analysis could not be performed. Therefore, no causation could be inferred from the findings. A further limitation was that health conditions and service use were self-reported. For some people, particularly those with dementia, there were some missing responses, but informant interviews and interviewer observations were used where possible to provide missing information. Nevertheless, there was still a certain level of non-response. There is also no way of knowing whether the increase in prevalence of diabetes seen was from a genuine increase in prevalence or whether it reflects a greater monitoring of the condition or increased awareness and diagnosis. For CFAS II, initial response rates to participate were substantially lower than for CFAS I; this is discussed fully elsewhere [17]. Analyses for both studies were weighted to account for non-response (and dropout for CFAS I) in order to minimise the risk of bias. Detailed analysis of CFAS II with respect to the prevalence of dementia revealed no considerable impact on results when making reasonable assumptions about response [10]. Out of scope of this analysis was the impact of co-occurring health conditions on the use of dementia services and this should be a focus of future work.

There was an overall increase in the use of services, particularly for day-to-day care workers, home help and day centres, by those with dementia and a health condition compared to having a health condition without dementia. Increased use of these services with increasing dementia severity has been previously reported [18, 19]. As overall numbers living in care homes have reduced, prevalence of both functional and cognitive impairment in residents has increased [20]. This could explain the pronounced association in CFAS II compared to CFAS I of those with a health condition and dementia using care workers, home help and day centres. With fewer residential care places available, pressure falls onto primary care teams and community-based care providers, such as care workers, home help and day centres, to help people with dementia remain in their homes for as long as possible. There was no increased use of GP services by individuals with dementia and another health condition versus the health condition alone, though this is possibly due to any increase in use of GP services being equal by all individuals, rather than a lack of increase.

Use of unpaid care was increased for people with dementia and another health condition in CFAS II but not in CFAS I, suggesting an increased need for care from friends and family now compared to in the past. The important role of family, friends and neighbours in providing dementia care has long been recognised [21–23]. However, such a caring role has been consistently shown to negatively affect the informal carer’s physical health and mental wellbeing [24]. A meta-analysis suggested that carer stress was only very weakly linked with premature institutionalisation, and that this literature showed evidence of severe bias, suggesting that patient cognitive and functional impairment were more important factors [25]. It is thus essential that the informal carer’s needs are considered by professional carers at the same time as those of the person with dementia [26]. A systematic review explored in detail the effects of combined carer/patient interventions on the health and wellbeing of both groups [27]. These programmes improved the general mental health of carers; however, effects on carer burden and coping, physical health and survival were less conclusive [27].

## Conclusion

In conclusion, there have been some changes in service use by individuals with dementia and another health condition over time, which could be indicative of the changes that have been seen with less people moving into care settings. More pressure is now being put on unpaid carers in dementia care specifically rather than with the care of other chronic conditions, and this pressure is likely to continue in the future. Access to home help, care workers and day centres should therefore be readily available as well as access to treatment and care for their own healthcare needs. However, an important aspect that we were unable to address in this study is the quality of chronic illness care. While the presence of dementia tended to increase service use; for example, we do not know if the quality of care received for diabetes is comparable for people with dementia to those without. Future research is necessary to examine whether care is optimum and in line with national guidelines.

## Additional file

Additional file 1: Age and sex adjusted estimates comparing service use of those with a health condition and dementia to service use of those with the health condition alone. Sensitivity analysis results also included. (DOCX 146 kb)

## Abbreviations

CI: Confidence interval
CFAS: Cognitive Function and Ageing Study
DSM IIIR: Diagnostic and Statistical Manual of Mental Disorders version III, revised
GMS: Geriatric Mental State
GP: General Practitioner
IRR: incident rate ratio
OR: odds ratio
