# Supplementary material for: The impact of dementia on service use by individuals with a comorbid health condition: a comparison of two cross-sectional analyses conducted approximately 10 years apart
Source: BMC Med. 2018 Jul 24;16:114. doi: 10.1186/s12916-018-1105-8 (PMC6056993; doi:10.1186/s12916-018-1105-8)
Supplement: Supplementary file 1 — Age and sex adjusted estimates comparing service use of those with a health condition and dementia to service use of those with the health condition alone. Sensitivity analysis results also included. (DOCX 146 kb) [file 12916_2018_1105_MOESM1_ESM.docx]

**Appendix**

**Adjusted for age and sex**

**Stroke**

**Table S1:** Risk ratio and 95% confidence interval comparing stroke and dementia to stroke by itself, adjusted for age and sex. Any services which did not have the numbers to calculate risk in either CFAS I or CFAS II were omitted from the table, ‘.’ Represents unable to model risk in one of the studies. See methods for description of weights.

|  | **CFAS I** | | **CFAS II** | |
| --- | --- | --- | --- | --- |
| **Service Counts** | IRR | 95% CI | IRR | 95% CI |
| Any Day to Day Service (count) | 2·0 | 1·3 – 3·1 | 1·3 | 0·8 – 1·9 |
| Any Service in prior four weeks (count) | 1·1 | 0·7 – 1·6 | 1·6 | 1·1 – 2·3 |
| Outpatient in last 3 months (count) | 0·6 | 0·1 – 2·4 | 0·4 | 0·2 – 1·0 |
| Day patient in last year (count) | · | · | 1·4 | 0·6 – 3·4 |
| Inpatient in last year (count) | 3·5 | 1·4 – 9·0 | 1·9 | 1·2 – 3·1 |
| **Individual services** | OR | 95% CI | OR | 95% CI |
| **Day to day services** |  |  |  |  |
| Home help | 2·8 | 0·4 – 18·0 | 1·1 | 0·2 – 6·0 |
| Care Worker | 5·5 | 1·9 – 16·1 | 9·3 | 3·8 – 22·5 |
| Warden | · | · | 1·0 | 0·1 – 9·0 |
| Paid Help | 1·4 | 0·3 – 7·5 | 0·3 | 0·1 – 1·2 |
| Any professional service | 4·1 | 1·2 – 13·7 | 2·1 | 1·0 – 4·3 |
| Unpaid Care | 1·2 | 0·4 – 3·6 | 1·9 | 0·9 – 4·3 |
| **Services in prior four weeks** |  |  |  |  |
| Home help/home care assistant | 4·1 | 1·3 – 12·8 | 5·8 | 2·5 – 13·6 |
| Any nursing Services | 0·7 | 0·2 – 2·6 | 2·2 | 0·9 – 5·2 |
| Chiropodist | 0·4 | 0·1 – 1·8 | 2·1 | 1·0 – 4·2 |
| Meals on wheels | 7·2 | 2·1 – 24·8 | · | · |
| Day Centre | 2·4 | 0·5 – 11·2 | 10·0 | 4·0 – 25·1 |
| GP | 0·6 | 0·2 – 2·2 | 1·1 | 0·5 – 2·4 |
| Any service | 0·6 | 0·2 – 1·8 | 0·8 | 0·4 – 1·7 |
| **Hospital Services** |  |  |  |  |
| Casualty/Outpatient | 0·9 | 0·2 – 4·5 | 0·6 | 0·2 – 1·8 |
| Day patient | · | · | 0·9 | 0·4 – 2·4 |
| Inpatient | 4·9 | 1·4 – 17·7 | 2·5 | 1·2 – 5·4 |

**Diabetes**

**Table S2:** Risk ratio and 95% confidence interval comparing diabetes and dementia to diabetes by itself, adjusted for age and sex. Any services which did not have the numbers to calculate risk in either CFAS I or CFAS II were omitted from the table, ‘.’ Represents unable to model risk in one of the studies. See methods for description of weights.

|  | **CFAS I** | | **CFAS II** | |
| --- | --- | --- | --- | --- |
| **Service counts** | IRR | 95% CI | IRR | 95% CI |
| Any Day to Day Service (count) | 1·2 | 0·6 – 2·2 | 1·1 | 0·7 – 1·8 |
| Any Service in prior four weeks (count) | 1·0 | 0·6 – 1·7 | 1·4 | 0·9 – 2·2 |
| Outpatient in last 3 months (count) | 0·5 | 0·2 – 1·4 | 0·6 | 0·3 – 1·2 |
| Day patient in last year (count) | 0·4 | 0·1 – 2·7 | 1·0 | 0·2 – 4·3 |
| Inpatient in last year (count) | 1·3 | 0·5 – 3·6 | 0·9 | 0·5 – 1·6 |
| **Individual Services** | OR | 95% CI | OR | 95% CI |
| **Day to day services** |  |  |  |  |
| Home help | · | · | 2·1 | 0·5 – 9·7 |
| Care Worker | 1·8 | 0·5 – 6·8 | 5·0 | 1·8 – 14·1 |
| Warden | · | · | 0·8 | 0·1 – 7·1 |
| Paid Help | 0·8 | 0·1 – 7·1 | 0·1 | 0·0 – 0·8 |
| Other | · | · | 1·1 | 0·1 – 9·6 |
| Any professional day to day service | 1·9 | 0·5 – 6·3 | 1·4 | 0·7 – 3·1 |
| Unpaid Care | 0·9 | 0·3 – 2·8 | 2·8 | 1·3 – 5·9 |
| **Services in prior four weeks** |  |  |  |  |
| Home help/Home care assistant | 1·3 | 0·3 – 5·6 | 3·7 | 1·4 – 9·4 |
| Any nursing service | 1·3 | 0·4 – 4·4 | 2·4 | 1·1 – 5·4 |
| Chiropodist | 0·4 | 0·1 – 1·5 | 0·9 | 0·4 – 2·0 |
| Meals on Wheels | 1·6 | 0·4 – 6·8 | 0·6 | 0·1 – 5·7 |
| Occupational therapist | · | · | 2·1 | 0·2 – 18·6 |
| Day Centre | 2·8 | 0·6 – 12·6 | 6·8 | 2·2 – 21·0 |
| GP | 1·0 | 0·3 – 3·5 | 0·9 | 0·4 – 1·8 |
| Any Service | 0·6 | 0·1 – 2·4 | 1·0 | 0·5 – 2·2 |
| **Hospital Services** |  |  |  |  |
| Casualty/outpatient | 0·3 | 0·1 – 1·4 | 0·9 | 0·4 – 2·4 |
| Day patient | 0·5 | 0·1 – 4·1 | 0·4 | 0·1 – 1·4 |
| Inpatient | 1·4 | 0·4 – 4·8 | 1·3 | 0·5 – 3·0 |

**Visual Impairment**

**Table S3:** Risk ratio and 95% confidence interval comparing visual impairment and dementia to visual impairment by itself, adjusted for age and sex. Any services which did not have the numbers to calculate risk in either CFAS I or CFAS II were omitted from the table, ‘.’ Represents unable to model risk in one of the studies. See methods for description of weights.

|  | **CFAS I** | | **CFAS II** | |
| --- | --- | --- | --- | --- |
| **Service Counts** | IRR | 95% CI | IRR | 95% CI |
| Any Day to Day Service (count) | 1·5 | 0·9 – 2·6 | 1·1 | 0·7 – 1·7 |
| Any Service in prior four weeks (count) | 0·9 | 0·6 – 1·6 | 1·3 | 1·0 – 1·6 |
| Outpatient in last 3 months (count) | 0·9 | 0·3 – 3·0 | 0·6 | 0·3 – 1·1 |
| Day patient in last year (count) | 0·4 | 0·1 – 2·7 | 0·8 | 0·3 – 2·1 |
| Inpatient in last year (count) | 3·5 | 1·1 – 11·5 | 1·7 | 0·9 – 3·2 |
| **Individual Services** | OR | 95% CI | OR | 95% CI |
| **Day to day services** |  |  |  |  |
| Home help | 2·1 | 0·2 – 19·4 | 0·5 | 0·1 – 4·4 |
| Care Worker | 5·8 | 1·8 – 19·2 | 6·4 | 2·6 – 15·5 |
| Warden | · | · | 0·9 | 0·1 – 7·8 |
| Paid Help | 0·2 | 0·0 – 1·9 | 0·1 | 0·0 – 0·7 |
| Any professional day to day service | 2·0 | 0·7 – 5·9 | 1·6 | 0·7 – 3·7 |
| Unpaid Care | 0·9 | 0·3 – 2·5 | 2·9 | 1·3 – 6·7 |
| **Services in prior four weeks** |  |  |  |  |
| Home help/Home care assistant | 4·4 | 1·3 – 15·0 | 3·4 | 1·3 – 8·5 |
| Any nursing service | 0·5 | 0·1 – 1·9 | 1·0 | 0·4 – 2·4 |
| Chiropodist | 0·5 | 0·1 – 2·5 | 1·8 | 0·9 – 3·8 |
| Meals on Wheels | 7·5 | 2·0 – 28·3 | 0·8 | 0·1 – 6·6 |
| Day Centre | · | · | 6·5 | 2·0 – 20·7 |
| GP | 0·4 | 0·1 – 1·6 | 1·1 | 0·5 – 2·2 |
| Any Service | 0·4 | 0·1 – 1·1 | 2·9 | 1·1 – 7·5 |
| **Hospital Services** |  |  |  |  |
| Casualty/outpatient | 1·9 | 0·4 – 8·5 | 0·7 | 0·3 – 1·5 |
| Day patient | 0·5 | 0·1 – 4·5 | 0·8 | 0·3 – 2·3 |
| Inpatient | 3·4 | 0·8 – 14·7 | 1·5 | 0·7 – 3·4 |

**Angina**

**Table S4:** Risk ratio and 95% confidence interval comparing angina and dementia to angina by itself, adjusted for age and sex. Any services which did not have the numbers to calculate risk in either CFAS I or CFAS II were omitted from the table, ‘.’ Represents unable to model risk in one of the studies. See methods for description of weights.

|  | **CFAS I** | | **CFAS II** | |
| --- | --- | --- | --- | --- |
| **Service Counts** | IRR | 95% CI | IRR | 95% CI |
| Any Day to Day Service (count) | 1·6 | 1·1 – 2·3 | 1·3 | 0·7 – 2·3 |
| Any Service in prior four weeks (count) | 1·4 | 1·0 – 1·8 | 1·0 | 0·7 – 1·3 |
| Outpatient in last 3 months (count) | 1·5 | 0·7 – 3·0 | 0·7 | 0·4 – 1·2 |
| Day patient in last year (count) | 0·3 | 0·0 – 1·9 | 0·2 | 0·1 – 0·8 |
| Inpatient in last year (count) | 3·3 | 1·4 – 7·9 | 0·9 | 0·5 – 1·6 |
| **Individual Services** | OR | 95% CI | OR | 95% CI |
| **Day to day services** |  |  |  |  |
| Home help | 0·5 | 0·1 – 4·1 | 1·3 | 0·2 – 6·6 |
| Care Worker | 10·4 | 3·5 – 30·8 | 5·3 | 2·1 – 13·7 |
| Warden | · | · | 1·7 | 0·4 – 8·5 |
| Paid Help | 0·1 | 0·0 – 1·3 | 0·4 | 0·2 – 1·3 |
| Any professional day to day service | 3·6 | 1·4 – 9·4 | 1·1 | 0·5 – 2·5 |
| Unpaid Care | 1·0 | 0·3 – 2·9 | 3·9 | 1·7 – 8·9 |
| **Services in prior four weeks** |  |  |  |  |
| Home help/Home care assistant | 8·7 | 3·1 – 25·1 | 4·0 | 1·7 – 9·3 |
| Any nursing service | 0·6 | 0·2 – 2·3 | 1·0 | 0·5 – 2·2 |
| Chiropodist | 1·2 | 0·3 – 4·5 | 0·5 | 0·2 – 1·2 |
| Meals on wheels | 4·9 | 1·3 – 17·9 | . | . |
| Physiotherapy | · | · | 0·7 | 0·2 – 3·1 |
| Occupational therapy | · | · | 5·7 | 1·0 – 33·6 |
| Day Centre | 0·9 | 0·2 – 5·1 | 3·7 | 1·1 – 12·8 |
| GP | 0·4 | 0·1 – 1·3 | 0·7 | 0·4 – 1·4 |
| Any Service | 1·9 | 0·6 – 5·5 | 1·0 | 0·4 – 2·1 |
| **Hospital Services** |  |  |  |  |
| Casualty/outpatient | 2·1 | 0·6 – 7·3 | 0·7 | 0·4 – 1·6 |
| Day patient | 0·3 | 0·0 – 2·8 | 0·2 | 0·1 – 1·0 |
| Inpatient | 3·9 | 1·2 – 13·2 | 0·9 | 0·5 – 1·9 |

**Parkinson’s disease**

**Table S5:** Risk ratio and 95% confidence interval comparing Parkinson’s disease and dementia to Parkinson’s disease by itself, adjusted for age and sex. Any services which did not have the numbers to calculate risk in either CFAS I or CFAS II were omitted from the table, ‘.’ Represents unable to model risk in one of the studies. See methods for description of weights.

|  | **CFAS I** | | **CFAS II** | |
| --- | --- | --- | --- | --- |
| **Service Counts** | IRR | 95% CI | IRR | 95% CI |
| Any Day to Day Service (count) | 0·5 | 0·1 – 2·5 | 1·1 | 0·3 – 4·0 |
| Any Service in prior four weeks (count) | 1·0 | 0·4 – 2·7 | 0·8 | 0·4 – 1·4 |
| Outpatient in last 3 months (count) | 0·9 | 0·2 – 3·7 | 0·3 | 0·1 – 2·2 |
| Day patient in last year (count) | · | · | · | · |
| Inpatient in last year (count) | · | · | 0·8 | 0·1 – 5·3 |
| **Individual Services** | OR | 95% CI | OR | 95% CI |
| **Day to day services** |  |  |  |  |
| Home help | · | · | · | · |
| Care Worker | · | · | · | · |
| Warden | · | · | · | · |
| Paid Help | · | · | 0·5 | 0·0 – 7·4 |
| Any professional day to day service | 0·5 | 0·0 – 6·6 | 1·1 | 0·1 – 9·5 |
| Unpaid Care | 0·2 | 0·0 – 4·5 | 2·5 | 0·2 – 28·4 |
| **Services in prior four weeks** |  |  |  |  |
| Home help/Home care assistant | 2·2 | 0·1 – 49·7 | 4·6 | 0·5 – 40·7 |
| Any nursing service | 0·9 | 0·0 – 18·6 | 0·8 | 0·1 – 4·8 |
| Chiropodist | 0·3 | 0·0 – 5·4 | 0·4 | 0·1 – 3·2 |
| Physiotherapy | · | · | 0·6 | 0·1 – 6·9 |
| Day Centre | · | · | · | · |
| GP | · | · | · | · |
| Any Service | 0·3 | 0·0 – 4·9 | 1·2 | 0·1 – 12·5 |
| **Hospital Services** |  |  |  |  |
| Casualty/outpatient | 1·4 | 0·1 – 33·8 | 0·4 | 0·0 – 3·5 |
| Day patient | · | · | · | · |
| Inpatient | · | · | 0·8 | 0·1 – 7·0 |

**Hypertension**

**Table S6:** Risk ratio and 95% confidence interval comparing hypertension and dementia to hypertension by itself, adjusted for age and sex. Any services which did not have the numbers to calculate risk in either CFAS I or CFAS II were omitted from the table, ‘.’ Represents unable to model risk in one of the studies. See methods for description of weights.

|  | **CFAS I** | | **CFAS II** | |
| --- | --- | --- | --- | --- |
| **Service Counts** | IRR | 95% CI | IRR | 95% CI |
| Any Day to Day Service (count) | 1·5 | 1·0 – 2·3 | 1·2 | 0·8 – 1·8 |
| Any Service in prior four weeks (count) | 1·1 | 0·9 – 1·5 | 1·0 | 0·8 – 1·3 |
| Outpatient in last 3 months (count) | 0·5 | 0·2 – 1·4 | 0·6 | 0·4 – 0·9 |
| Day patient in last year (count) | · | · | 0·5 | 0·2 – 1·4 |
| Inpatient in last year (count) | 2·6 | 1·1 – 6·2 | 1·0 | 0·6 – 1·6 |
| **Individual Services** | OR | 95% CI | OR | 95% CI |
| **Day to day services** |  |  |  |  |
| Home help | 1·5 | 0·3 – 7·3 | 0·5 | 0·1 – 2·4 |
| Care Worker | 3·7 | 1·6 – 8·7 | 6·5 | 3·4 – 12·2 |
| Meals on wheels | 6·7 | 1·4 – 31·5 | 1·4 | 0·2 – 10·4 |
| Community nurse | · | · | 1·5 | 0·2 – 13·7 |
| Warden | · | · | 1·5 | 0·3 – 6·6 |
| Paid Help | 0·6 | 0·2 – 2·1 | 0·5 | 0·2 – 1·1 |
| Other professional services | · | · | 0·8 | 0·1 – 6·2 |
| Any professional day to day service | 2·2 | 1·0 – 4·6 | 1·1 | 0·7 – 1·9 |
| Unpaid Care | 2·0 | 0·9 – 4·5 | 3·2 | 1·9 – 5·4 |
| **Services in prior four weeks** |  |  |  |  |
| Home help/Home care assistant | 1·5 | 0·5 – 4·2 | 4·9 | 2·8 – 8·6 |
| Any nursing service | 1·3 | 0·6 – 2·9 | 1·5 | 0·9 – 2·5 |
| Chiropodist | 0·5 | 0·2 – 1·3 | 0·9 | 0·6 – 1·6 |
| Meals on Wheels | 3·5 | 1·3 – 9·4 | 1·5 | 0·4 – 5·4 |
| Physiotherapist | 3·3 | 0·5 – 21·6 | 0·8 | 0·3 – 2·3 |
| Occupational therapist | · | · | 2·5 | 0·7 – 9·2 |
| Speech therapist | · | · | 1·4 | 0·2 – 13·1 |
| Social worker | 1·3 | 0·2 – 11·2 | 1·6 | 0·2 – 14·9 |
| Day Centre | 2·4 | 0·8 – 7·4 | 7·0 | 3·5 – 13·9 |
| GP | 0·8 | 0·4 – 1·7 | 1·1 | 0·7 – 1·7 |
| Any Service | 1·1 | 0·5 – 2·4 | 0·8 | 0·5 – 1·3 |
| **Hospital Services** |  |  |  |  |
| Casualty/outpatient | 0·8 | 0·3 – 2·3 | 0·8 | 0·5 – 1·3 |
| Day patient | · | · | 0·5 | 0·2 – 1·1 |
| Inpatient | 2·1 | 0·8 – 5·4 | 0·9 | 0·5 – 1·5 |

**Anaemia**

**Table S7:** Risk ratio and 95% confidence interval comparing anaemia and dementia to anaemia by itself, adjusted for age and sex. Any services which did not have the numbers to calculate risk in either CFAS I or CFAS II were omitted from the table, ‘.’ Represents unable to model risk in one of the studies. See methods for description of weights.

|  | **CFAS I** | | **CFAS II** | |
| --- | --- | --- | --- | --- |
| **Service Counts** | IRR | 95% CI | IRR | 95% CI |
| Any Day to Day Service (count) | 2·4 | 1·4 – 4·1 | 1·0 | 0·4 – 2·5 |
| Any Service in prior four weeks (count) | 1·4 | 0·9 – 2·1 | 0·9 | 0·4 – 1·7 |
| Outpatient in last 3 months (count) | · | · | 0·3 | 0·1 – 0·9 |
| Day patient in last year (count) | 0·4 | 0·1 – 2·3 | 0·7 | 0·4 – 1·4 |
| Inpatient in last year (count) | 0·5 | 0·1 – 2·0 | 1·3 | 0·3 – 4·9 |
| **Individual Services** | OR | 95% CI | OR | 95% CI |
| **Day to day services** |  |  |  |  |
| Home help | · | · | · | · |
| Care Worker | 4·8 | 0·9 – 25·0 | 8·0 | 1·5 – 41·5 |
| Warden | · | · | · | · |
| Paid Help | · | · | · | · |
| Any professional day to day service | 9·2 | 2·0 – 42·0 | 1·0 | 0·3 – 4·0 |
| Unpaid Care | 5·9 | 0·7 – 47·0 | 1·8 | 0·5 – 6·9 |
| **Services in prior four weeks** |  |  |  |  |
| Home help/Home care assistant | 9·6 | 2·6 – 35·9 | 2·6 | 0·5 – 12·8 |
| Any nursing service | 0·4 | 0·1 – 2·6 | 3·2 | 1·0 – 10·4 |
| Chiropodist | 2·6 | 0·7 – 10·2 | 1·5 | 0·4 – 6·4 |
| Meals on wheels | 1·7 | 0·2 – 15·6 |  |  |
| Physiotherapist | · | · | 1·2 | 0·1 – 10·5 |
| Occupational therapist | · | · | 3·9 | 0·3 – 44·5 |
| Social worker | 2·7 | 0·3 – 28·1 | · | · |
| Day Centre | 2·6 | 0·4 – 18·5 | 4·8 | 0·7 – 34·8 |
| Day hospital | · | · | 2·1 | 0·2 – 19·5 |
| GP | 0·8 | 0·2 – 3·4 | 0·4 | 0·1 – 1·5 |
| Any Service | 2·2 | 0·3 – 18·4 | 0·5 | 0·1 – 1·7 |
| **Hospital Services** |  |  |  |  |
| Casualty/outpatient | 0·2 | 0·0 – 1·9 | 0·5 | 0·1 – 2·0 |
| Day patient | 0·6 | 0·1 – 5·8 | 1·6 | 0·4 – 6·8 |
| Inpatient | 0·8 | 0·1 – 4·7 | 0·6 | 0·1 – 2·9 |

**Breathing difficulties**

**Table S8:** Risk ratio and 95% confidence interval comparing breathing difficulties and dementia to breathing difficulties by itself, adjusted for age and sex. Any services which did not have the numbers to calculate risk in either CFAS I or CFAS II were omitted from the table, ‘.’ Represents unable to model risk in one of the studies. See methods for description of weights.

|  | **CFAS I** | | **CFAS II** | |
| --- | --- | --- | --- | --- |
| **Service Counts** | IRR | 95% CI | IRR | 95% CI |
| Any Day to Day Service (count) | 1·0 | 0·6 – 1·6 | 1·2 | 0·7 – 2·3 |
| Any Service in prior four weeks (count) | 1·1 | 0·8 – 1·6 | 1·1 | 0·7 – 1·6 |
| Outpatient in last 3 months (count) | 0·8 | 0·3 – 1·9 | 0·5 | 0·3 – 1·1 |
| Day patient in last year (count) | · | · | 1·2 | 0·3 – 5·2 |
| Inpatient in last year (count) | 2·3 | 1·1 – 4·8 | 2·0 | 1·0 – 3·7 |
| **Individual Services** | OR | 95% CI | OR | 95% CI |
| **Day to day services** |  |  |  |  |
| Home help | · | · | 1·5 | 0·2 – 13·8 |
| Care Worker | 4·5 | 1·6 – 12·3 | 3·9 | 1·2 – 12·9 |
| Warden | · | · | 2·3 | 0·3 – 17·0 |
| Paid Help | 0·5 | 0·1 – 2·1 | 1·0 | 0·3 – 2·8 |
| Any professional day to day service | 1·5 | 0·6 – 3·7 | 1·3 | 0·5 – 3·1 |
| Unpaid Care | 0·9 | 0·3 – 2·4 | 2·1 | 1·0 – 4·7 |
| **Services in prior four weeks** |  |  |  |  |
| Home help/Home care assistant | 3·4 | 1·2 – 9·6 | 3·8 | 1·5 – 9·7 |
| Any nursing service | 2·2 | 0·8 – 6·0 | 2·8 | 1·2 – 6·5 |
| Chiropodist | 1·3 | 0·5 – 3·2 | 1·2 | 0·5 – 3·0 |
| Meals on wheels | 3·9 | 0·9 – 16·3 |  |  |
| Physiotherapist | · | · | 1·1 | 0·2 – 4·9 |
| Occupational therapist | · | · | 3·2 | 0·6 – 15·8 |
| Day Centre | 1·1 | 0·2 – 5·7 | 4·2 | 1·1 – 16·0 |
| Day Hospital | 3·8 | 0·6 – 23·8 |  |  |
| GP | 0·7 | 0·2 – 1·8 | 1·1 | 0·5 – 2·4 |
| Any Service | 0·8 | 0·3 – 2·0 | 0·7 | 0·3 – 1·4 |
| **Hospital Services** |  |  |  |  |
| Casualty/outpatient | 0·7 | 0·2 – 2·2 | 0·6 | 0·3 – 1·6 |
| Day patient | · | · | 0·7 | 0·2 – 2·6 |
| Inpatient | 2·6 | 1·0 – 6·9 | 1·6 | 0·7 – 3·5 |

**Transient ischaemic attack**

**Table S9:** Risk ratio and 95% confidence interval comparing transient ischaemic attack and dementia to transient ischaemic attack by itself, adjusted for age and sex. Any services which did not have the numbers to calculate risk in either CFAS I or CFAS II were omitted from the table, ‘.’ Represents unable to model risk in one of the studies. See methods for description of weights.

|  | **CFAS I** | | **CFAS II** | |
| --- | --- | --- | --- | --- |
| **Service Counts** | IRR | 95% CI | IRR | 95% CI |
| Any Day to Day Service (count) | 1·5 | 0·9 – 2·4 | 1·8 | 1·2 – 2·8 |
| Any Service in prior four weeks (count) | 1·1 | 0·8 – 1·6 | 1·2 | 0·9 – 1·6 |
| Outpatient in last 3 months (count) | 0·8 | 0·4 – 1·7 | 0·5 | 0·3 – 1·0 |
| Day patient in last year (count) | 0·3 | 0·1 – 1·3 | 0·8 | 0·5 – 1·4 |
| Inpatient in last year (count) | 3·4 | 1·6 – 6·9 | 1·4 | 0·8 – 2·5 |
| **Individual Services** | OR | 95% CI | OR | 95% CI |
| **Day to day services** |  |  |  |  |
| Home help | 2·0 | 0·5 – 8·1 | 1·6 | 0·3 – 9·8 |
| Care Worker | 4·5 | 1·8 – 11·2 | 7·6 | 3·3 – 17·6 |
| Community nurse | · | · | 3·5 | 0·3 – 35·5 |
| Warden | · | · | 9·4 | 1·5 – 58·4 |
| Paid Help | 0·7 | 0·2 – 2·8 | 0·8 | 0·3 – 2·2 |
| Other professional services | · | · | 0·9 | 0·1 – 7·2 |
| Any professional day to day service | 1·7 | 0·8 – 4·0 | 2·2 | 1·0 – 4·5 |
| Unpaid Care | 0·9 | 0·4 – 1·9 | 2·2 | 1·0 – 4·8 |
| **Services in prior four weeks** |  |  |  |  |
| Home help/Home care assistant | 3·5 | 1·4 – 8·8 | 4·6 | 1·9 – 10·7 |
| Any nursing service | 1·2 | 0·5 – 2·9 | 1·5 | 0·7 – 3·2 |
| Chiropodist | 0·7 | 0·3 – 2·1 | 1·0 | 0·5 – 2·2 |
| Meals on Wheels | 3·0 | 0·9 – 9·9 | 1·7 | 0·2 – 18·3 |
| Physiotherapist | 4·6 | 0·6 – 33·1 | 1·0 | 0·3 – 3·2 |
| Occupational therapist | · | · | 4·8 | 0·7 – 30·7 |
| Speech therapist | · | · | 1·4 | 0·1 – 18·4 |
| Social worker | 1·0 | 0·1 – 9·7 | 1·8 | 0·1 – 21·8 |
| Day Centre | 1·7 | 0·5 – 5·7 | 7·2 | 2·6 – 19·8 |
| Day hospital | 5·5 | 0·8 – 37·8 | 0·8 | 0·1 – 7·1 |
| GP | 1·1 | 0·5 – 2·7 | 1·1 | 0·6 – 2·0 |
| Any Service | 0·5 | 0·2 – 1·1 | 1·2 | 0·6 – 2·6 |
| **Hospital Services** |  |  |  |  |
| Casualty/outpatient | 1·3 | 0·5 – 3·5 | 0·6 | 0·3 – 1·2 |
| Day patient | 0·5 | 0·1 – 2·1 | 1·3 | 0·6 – 2·8 |
| Inpatient | 3·6 | 1·4 – 9·0 | 1·4 | 0·7 – 2·8 |

**Heart attack**

**Table S10:** Risk ratio and 95% confidence interval comparing heart attack and dementia to heart attack by itself, adjusted for age and sex. Any services which did not have the numbers to calculate risk in either CFAS I or CFAS II were omitted from the table, ‘.’ Represents unable to model risk in one of the studies. See methods for description of weights.

|  | **CFAS I** | | **CFAS II** | |
| --- | --- | --- | --- | --- |
| **Service Counts** | IRR | 95% CI | IRR | 95% CI |
| Any Day to Day Service (count) | 1·1 | 0·5 – 2·2 | 1·4 | 0·8 – 2·5 |
| Any Service in prior four weeks (count) | 1·0 | 0·6 – 1·6 | 1·1 | 0·8 – 1·6 |
| Outpatient in last 3 months (count) | 1·0 | 0·3 – 2·8 | 0·3 | 0·2 – 0·7 |
| Day patient in last year (count) | · | · | 1·7 | 0·7 – 3·8 |
| Inpatient in last year (count) | 2·8 | 1·0 – 7·4 | 1·0 | 0·6 – 1·9 |
| **Individual Services** | OR | 95% CI | OR | 95% CI |
| **Day to day services** |  |  |  |  |
| Home help | · | · | 2·0 | 0·5 – 8·2 |
| Care Worker | 3·5 | 1·0 – 12·9 | 11·7 | 4·4 – 30·8 |
| Warden | · | · | 2·7 | 0·3 – 26·0 |
| Paid Help | 0·2 | 0·0 – 1·7 | 0·4 | 0·1 – 1·3 |
| Any professional day to day service | 1·0 | 0·3 – 3·2 | 1·3 | 0·6 – 2·9 |
| Unpaid Care | 0·5 | 0·1 – 1·6 | 2·6 | 1·1 – 6·0 |
| **Services in prior four weeks** |  |  |  |  |
| Home help/Home care assistant | 2·2 | 0·6 – 8·1 | 5·3 | 2·2 – 12·8 |
| Any nursing service | 0·6 | 0·1 – 2·8 | 0·9 | 0·4 – 2·1 |
| Chiropodist | 1·0 | 0·2 – 4·9 | 0·9 | 0·4 – 2·1 |
| Meals on wheels | 3·8 | 0·7 – 19·9 | · | · |
| Physiotherapist | · | · | 0·4 | 0·0 – 2·8 |
| Day Centre | · | · | 5·9 | 1·7 – 21·1 |
| Day hospital | 2·8 | 0·2 – 45·9 | 2·7 | 0·6 – 13·2 |
| GP | 0·4 | 0·1 – 1·6 | 1·2 | 0·6 – 2·4 |
| Any Service | 0·8 | 0·3 – 2·3 | 1·1 | 0·5 – 2·5 |
| **Hospital Services** |  |  |  |  |
| Casualty/outpatient | 1·4 | 0·3 – 7·2 | 0·5 | 0·2 – 1·1 |
| Day patient | · | · | 0·4 | 0·2 – 1·3 |
| Inpatient | 2·5 | 0·6 – 10·9 | 0·9 | 0·4 – 1·8 |

**Hearing difficulties**

**Table S11:** Risk ratio and 95% confidence interval comparing hearing difficulties and dementia to hearing difficulties by itself, adjusted for age and sex. Any services which did not have the numbers to calculate risk in either CFAS I or CFAS II were omitted from the table, ‘.’ Represents unable to model risk in one of the studies. See methods for description of weights.

|  | **CFAS I** | | **CFAS II** | |
| --- | --- | --- | --- | --- |
| **Service Counts** | IRR | 95% CI | IRR | 95% CI |
| Any Day to Day Service (count) | 0·9 | 0·5 – 1·9 | 1·2 | 0·8 – 1·7 |
| Any Service in prior four weeks (count) | 0·8 | 0·5 – 1·4 | 1·0 | 0·8 – 1·3 |
| Outpatient in last 3 months (count) | 0·5 | 0·1 – 1·9 | 0·6 | 0·4 – 0·9 |
| Day patient in last year (count) | 0·5 | 0·1 – 3·3 | 0·6 | 0·3 – 1·3 |
| Inpatient in last year (count) | 1·6 | 0·6 – 3·8 | 0·9 | 0·6 – 1·5 |
| **Individual Services** | OR | 95% CI | OR | 95% CI |
| **Day to day services** |  |  |  |  |
| Home help | 1·9 | 0·3 – 11·2 | 1·3 | 0·5 – 3·7 |
| Care Worker | 2·4 | 0·6 – 9·8 | 5·4 | 2·8 – 10·4 |
| Meals on wheels | · | · | 1·1 | 0·1 – 8·7 |
| Community worker | · | · | 4·1 | 0·4 – 40·2 |
| Community nurse | · | · | 1·0 | 0·1 – 8·5 |
| Warden | · | · | 1·6 | 0·4 – 6·1 |
| Paid Help | 0·6 | 0·1 – 4·0 | 0·4 | 0·2 – 0·8 |
| Any professional day to day service | 1·1 | 0·4 – 3·5 | 1·4 | 0·8 – 2·4 |
| Unpaid Care | 0·9 | 0·3 – 2·8 | 2·1 | 1·3 – 3·6 |
| **Services in prior four weeks** |  |  |  |  |
| Home help/Home care assistant | 1·7 | 0·4 – 6·7 | 5·2 | 2·9 – 9·4 |
| Any nursing service | 0·3 | 0·1 – 2·0 | 1·3 | 0·7 – 2·1 |
| Chiropodist | 0·9 | 0·3 – 2·7 | 1·0 | 0·6 – 1·8 |
| Meals on Wheels | 1·7 | 0·3 – 9·4 | 1·3 | 0·3 – 4·6 |
| Physiotherapist | · | · | 0·3 | 0·1 – 1·4 |
| Occupational therapist | · | · | 0·9 | 0·1 – 6·8 |
| Day Centre | 1·6 | 0·3 - 9·6 | 6·3 | 2·6 – 15·4 |
| Day hospital | · | · | 4·9 | 1·4 – 17·4 |
| GP | 0·9 | 0·3 – 2·8 | 0·8 | 0·5 – 1·3 |
| Any Service | 0·4 | 0·1 – 1·0 | 0·8 | 0·5 – 1·3 |
| **Hospital Services** |  |  |  |  |
| Casualty/outpatient | 0·5 | 0·1 – 2·2 | 0·9 | 0·5 – 1·4 |
| Day patient | 0·7 | 0·1 – 5·5 | 0·7 | 0·3 – 1·4 |
| Inpatient | 1·8 | 0·5 – 5·8 | 0·9 | 0·5 – 1·5 |

**Any target comorbidity**

**Table S12:** Risk ratio and 95% confidence interval comparing any target comorbidity and dementia to any target comorbidity by itself, adjusted for age and sex. Any services which did not have the numbers to calculate risk in either CFAS I or CFAS II were omitted from the table, ‘.’ Represents unable to model risk in one of the studies. See methods for description of weights.

|  | **CFAS I** | | **CFAS II** | |
| --- | --- | --- | --- | --- |
| **Service Counts** | IRR | 95% CI | IRR | 95% CI |
| Any Day to Day Service (count) | 1·6 | 1·1 – 2·4 | 1·1 | 0·8 – 1·6 |
| Any Service in prior four weeks (count) | 1·1 | 0·8 – 1·5 | 1·4 | 1·1 – 1·8 |
| Outpatient in last 3 months (count) | 0·5 | 0·2 – 1·2 | 0·5 | 0·3 – 0·8 |
| Day patient in last year (count) | 0·1 | 0·0 – 1·0 | 0·9 | 0·4 – 2·1 |
| Inpatient in last year (count) | 2·6 | 1·3 – 5·5 | 1·4 | 0·9 – 2·2 |
| **Individual Services** | OR | 95% CI | OR | 95% CI |
| **Day to day services** |  |  |  |  |
| Home help | 1·6 | 0·3 – 8·1 | 1·5 | 0·5 – 4·4 |
| Care worker | 4·2 | 1·9 – 9·0 | 5·6 | 3·0 – 10·4 |
| Community nurse | · | · | 4·4 | 0·9 – 22·4 |
| Warden | · | · | 0·5 | 0·1 – 3·8 |
| Paid help | 0·8 | 0·2 – 2·6 | 0·2 | 0·1 – 0·6 |
| Other day to day services | · | · | 0·7 | 0·1 – 5·7 |
| Any service | 2·0 | 1·0 – 4·3 | 1·5 | 0·9 – 2·6 |
| Unpaid care | 0·8 | 0·4 – 1·6 | 2·7 | 1·6 – 4·5 |
| **Services in prior four weeks** |  |  |  |  |
| Home help/home care assistant | 2·9 | 1·2 – 6·7 | 4·1 | 2·3 – 7·6 |
| Any nursing Services | 1·2 | 0·5 – 2·6 | 2·0 | 1·1 – 3·5 |
| Chiropodist | 0·5 | 0·2 – 1·4 | 1·6 | 1·0 – 2·7 |
| Meals on wheels | 5·3 | 2·2 – 12·8 | 0·4 | 0·0 – 3·2 |
| Physiotherapist | 4·5 | 0·6 – 36·0 | 0·6 | 0·2 – 2·1 |
| Occupational Therapist | · | · | 2·3 | 0·6 – 8·6 |
| Day Centre | 1·8 | 0·6 – 5·5 | 7·2 | 3·4 – 15·1 |
| Day Hospital | 7·2 | 1·4 – 37·2 | 5·2 | 1·5 – 17·8 |
| GP | 0·9 | 0·4 – 1·9 | 1·1 | 0·7 – 1·7 |
| Any service | 0·5 | 0·2 – 1·0 | 1·2 | 0·8 – 2·0 |
| **Hospital Services** |  |  |  |  |
| Casualty/Outpatient | 0·8 | 0·3 – 2·1 | 0·6 | 0·3 – 1·1 |
| Day patient | 0·2 | 0·0 – 1·3 | 0·6 | 0·3 – 1·2 |
| Inpatient | 3·1 | 1·3 – 7·1 | 1·5 | 0·9 – 2·6 |

**Adjusted for deprivation**

**Stroke**

**Table S13:** Risk ratio and 95% confidence interval comparing stroke and dementia to stroke by itself, adjusted for deprivation. Any services which did not have the numbers to calculate risk in either CFAS I or CFAS II were omitted from the table, ‘.’ Represents unable to model risk in one of the studies. See methods for description of weights.

|  | **CFAS I** | | **CFAS II** | |
| --- | --- | --- | --- | --- |
| **Service Counts** | IRR | 95% CI | IRR | 95% CI |
| Any Day to Day Service (count) | 2.8 | 1.8 – 4.5 | 1.6 | 1.0 – 2.4 |
| Any Service in prior four weeks (count) | 1.2 | 0.8 – 1.9 | 1.7 | 1.1 – 2.6 |
| Outpatient in last 3 months (count) | 0.6 | 0.2 – 2.1 | 0.4 | 0.2 – 0.9 |
| Day patient in last year (count) | . | . | 1.5 | 0.7 – 3.3 |
| Inpatient in last year (count) | 3.4 | 1.3 – 8.8 | 2.1 | 1.3 – 3.3 |
| **Individual services** | OR | 95% CI | OR | 95% CI |
| **Day to day services** |  |  |  |  |
| Home help | 3.8 | 0.7 – 21.7 | 1.2 | 0.2 – 5.8 |
| Care Worker | 6.5 | 2.0 – 21.0 | 9.4 | 4.0 – 22.1 |
| Warden | . | . | 1.3 | 0.1 – 1.2 |
| Paid Help | 2.0 | 0.6 – 7.2 | 0.4 | 0.1 – 1.3 |
| Any professional service | 5.5 | 1.7 – 17.2 | 2.4 | 1.2 – 4.9 |
| Unpaid Care | 1.5 | 0.5 – 4.8 | 2.1 | 1.0 – 4.4 |
| **Services in prior four weeks** |  |  |  |  |
| Home help/home care assistant | 5.2 | 1.5 – 18.1 | 5.9 | 2.5 – 13.8 |
| Any nursing Services | 0.7 | 0.2 – 2.5 | 2.3 | 0.9 – 5.5 |
| Chiropodist | 0.5 | 0.1 – 1.9 | 2.1 | 1.0 – 4.4 |
| Meals on wheels | 8.6 | 2.2 – 34.0 | . | . |
| Day Centre | 3.1 | 0.8 – 11.5 | 10.1 | 3.8 – 26.6 |
| GP | 0.6 | 0.2 – 2.1 | 1.2 | 0.5 – 2.5 |
| Any service | 0.7 | 0.2 – 2.3 | 0.9 | 0.4 – 1.8 |
| **Hospital Services** |  |  |  |  |
| Casualty/Outpatient | 0.9 | 0.2 – 4.4 | 0.5 | 0.2 – 1.6 |
| Day patient | . | . | 0.9 | 0.4 – 2.5 |
| Inpatient | 4.7 | 1.4 – 16.4 | 2.6 | 1.2 – 5.6 |

**Diabetes**

**Table S14:** Risk ratio and 95% confidence interval comparing diabetes and dementia to diabetes by itself, adjusted for deprivation. Any services which did not have the numbers to calculate risk in either CFAS I or CFAS II were omitted from the table, ‘.’ Represents unable to model risk in one of the studies. See methods for description of weights.

|  | **CFAS I** | | **CFAS II** | |
| --- | --- | --- | --- | --- |
| **Service counts** | IRR | 95% CI | IRR | 95% CI |
| Any Day to Day Service (count) | 1.9 | 1.1 – 3.4 | 1.7 | 1.0 – 2.9 |
| Any Service in prior four weeks (count) | 1.2 | 0.7 – 2.0 | 1.6 | 1.0 – 2.7 |
| Outpatient in last 3 months (count) | 0.4 | 0.1 – 1.4 | 0.6 | 0.3 – 1.2 |
| Day patient in last year (count) | . | . | 1.3 | 0.3 – 6.2 |
| Inpatient in last year (count) | 1.3 | 0.4 – 4.3 | 1.0 | 0.6 – 1.9 |
| **Individual Services** | OR | 95% CI | OR | 95% CI |
| **Day to day services** |  |  |  |  |
| Home help | . | . | 3.6 | 0.9 – 13.9 |
| Care Worker | 3.6 | 0.9 – 3.9 | 8.5 | 2.9 – 24.7 |
| Warden | . | . | 2.0 | 0.2 – 17.4 |
| Paid Help | 1.7 | 0.2 – 12.9 | 0.2 | 0.0 – 1.3 |
| Other | . | . | 1.1 | 0.1 – 9.2 |
| Any professional day to day service | 4.1 | 1.2 – 14.7 | 2.1 | 1.0 – 4.6 |
| Unpaid Care | 1.3 | 0.3 – 4.9 | 3.5 | 1.6 – 7.5 |
| **Services in prior four weeks** |  |  |  |  |
| Home help/Home care assistant | 2.4 | 0.6 – 10.7 | 6.1 | 2.3 – 15.7 |
| Any nursing service | 1.4 | 0.4 – 5.2 | 2.9 | 1.3 – 6.4 |
| Chiropodist | 0.4 | 0.1 – 1.8 | 1.2 | 0.6 – 2.6 |
| Meals on Wheels | 2.5 | 0.6 – 11.1 | 1.0 | 0.1 – 7.9 |
| Occupational therapist | . | . | 2.1 | 0.2 – 17.7 |
| Day Centre | 5.3 | 1.1 – 25.3 | 8.2 | 2.6 – 25.6 |
| GP | 1.0 | 0.3 – 3.7 | 0.9 | 0.4 – 1.9 |
| Any Service | 0.9 | 0.2 – 5.2 | 1.2 | 0.6 – 2.6 |
| **Hospital Services** |  |  |  |  |
| Casualty/outpatient | 0.2 | 0.0 – 0.9 | 0.9 | 0.4 – 2.2 |
| Day patient | 0.6 | 0.1 – 5.3 | 0.4 | 0.1 – 1.4 |
| Inpatient | 1.2 | 0.3 – 5.0 | 1.4 | 0.6 – 3.5 |

**Visual Impairment**

**Table S15:** Risk ratio and 95% confidence interval comparing visual impairment and dementia to visual impairment by itself, adjusted for deprivation. Any services which did not have the numbers to calculate risk in either CFAS I or CFAS II were omitted from the table, ‘.’ Represents unable to model risk in one of the studies. See methods for description of weights.

|  | **CFAS I** | | **CFAS II** | |
| --- | --- | --- | --- | --- |
| **Service Counts** | IRR | 95% CI | IRR | 95% CI |
| Any Day to Day Service (count) | 1.9 | 1.0 – 3.8 | 1.4 | 0.9 – 2.2 |
| Any Service in prior four weeks (count) | 1.0 | 0.5 – 1.7 | 1.4 | 1.1 – 1.8 |
| Outpatient in last 3 months (count) | 1.0 | 0.3 – 3.0 | 0.6 | 0.3 – 1.1 |
| Day patient in last year (count) | . | . | 1.0 | 0.3 – 2.7 |
| Inpatient in last year (count) | 3.7 | 1.1 – 11.9 | 1.8 | 0.9 – 3.5 |
| **Individual Services** | OR | 95% CI | OR | 95% CI |
| **Day to day services** |  |  |  |  |
| Home help | 2.5 | 0.3 – 21.3 | 0.7 | 0.1 – 5.1 |
| Care Worker | 5.7 | 1.6 – 20.6 | 8.1 | 3.3 – 19.8 |
| Warden | . | . | 1.5 | 0.2 – 12.3 |
| Paid Help | 0.3 | 0.0 – 2.5 | 0.1 | 0.0 – 0.9 |
| Any professional day to day service | 2.4 | 0.8 – 6.9 | 2.0 | 0.9 – 4.4 |
| Unpaid Care | 0.9 | 0.3 – 2.7 | 3.2 | 1.4 – 7.1 |
| **Services in prior four weeks** |  |  |  |  |
| Home help/Home care assistant | 4.2 | 1.1 – 15.7 | 4.2 | 1.7 – 10.7 |
| Any nursing service | 0.4 | 0.1 – 1.7 | 1.2 | 0.5 – 2.8 |
| Chiropodist | 0.7 | 0.2 – 2.5 | 2.0 | 0.9 – 4.3 |
| Meals on Wheels | 6.9 | 1.6 – 29.9 | 1.0 | 0.1 – 7.7 |
| Day Centre | . | . | 6.7 | 2.2 – 20.6 |
| GP | 0.4 | 0.1 – 1.6 | 1.1 | 0.5 – 2.3 |
| Any Service | 0.4 | 0.1 – 1.3 | 3.3 | 1.3 – 8.2 |
| **Hospital Services** |  |  |  |  |
| Casualty/outpatient | 2.1 | 0.5 – 9.1 | 0.7 | 0.3 – 1.5 |
| Day patient | 0.5 | 0.1 – 4.4 | 0.9 | 0.3 – 2.5 |
| Inpatient | 3.4 | 0.9 – 13.7 | 1.7 | 0.8 – 3.7 |

**Angina**

**Table S16:** Risk ratio and 95% confidence interval comparing angina and dementia to angina by itself, adjusted for deprivation. Any services which did not have the numbers to calculate risk in either CFAS I or CFAS II were omitted from the table, ‘.’ Represents unable to model risk in one of the studies. See methods for description of weights.

|  | **CFAS I** | | **CFAS II** | |
| --- | --- | --- | --- | --- |
| **Service Counts** | IRR | 95% CI | IRR | 95% CI |
| Any Day to Day Service (count) | 2.9 | 1.8 – 4.8 | 1.9 | 1.1 – 3.2 |
| Any Service in prior four weeks (count) | 1.7 | 1.2 – 2.3 | 1.1 | 0.8 – 1.4 |
| Outpatient in last 3 months (count) | 1.5 | 0.8 – 2.8 | 0.6 | 0.4 – 1.1 |
| Day patient in last year (count) | . | . | 0.2 | 0.0 – 0.7 |
| Inpatient in last year (count) | 3.2 | 1.2 – 8.0 | 0.9 | 0.5 – 1.7 |
| **Individual Services** | OR | 95% CI | OR | 95% CI |
| **Day to day services** |  |  |  |  |
| Home help | 0.8 | 0.1 – 7.0 | 1.8 | 0.4 – 8.6 |
| Care Worker | . | . | 7.3 | 3.1 – 17.1 |
| Warden | . | . | 2.3 | 0.5 – 11.6 |
| Paid Help | 0.3 | 0.0 – 2.7 | 0.7 | 0.3 – 1.9 |
| Any professional day to day service | 8.3 | 2.6 – 26.0 | 1.7 | 0.8 – 3.4 |
| Unpaid Care | 1.4 | 0.4 – 4.5 | 4.8 | 2.2 – 10.3 |
| **Services in prior four weeks** |  |  |  |  |
| Home help/Home care assistant | . | . | 5.0 | 2.3 – 10.8 |
| Any nursing service | 0.4 | 0.1 – 1.9 | 1.1 | 0.5 – 2.4 |
| Chiropodist | 1.7 | 0.5 – 5.6 | 0.7 | 0.3 – 1.4 |
| Meals on wheels | 8.2 | 2.2 – 30.7 | . | . |
| Physiotherapy | . | . | 0.8 | 0.2 – 3.5 |
| Occupational therapy | . | . | 6.3 | 1.1 – 35.5 |
| Day Centre | 1.7 | 0.3 – 8.4 | 4.7 | 1.5 – 14.5 |
| GP | 0.4 | 0.1 – 1.2 | 0.7 | 0.4 – 1.4 |
| Any Service | 3.1 | 0.8 – 11.6 | 1.1 | 0.5 – 2.4 |
| **Hospital Services** |  |  |  |  |
| Casualty/outpatient | 2.2 | 0.6 – 7.8 | 0.7 | 0.3 – 1.5 |
| Day patient | 0.4 | 0.0 – 3.1 | 0.2 | 0.1 – 0.9 |
| Inpatient | 3.3 | 1.0 – 11.0 | 1.0 | 0.5 – 2.0 |

**Parkinson’s disease**

**Table S17:** Risk ratio and 95% confidence interval comparing Parkinson’s disease and dementia to Parkinson’s disease by itself, adjusted for deprivation. Any services which did not have the numbers to calculate risk in either CFAS I or CFAS II were omitted from the table, ‘.’ Represents unable to model risk in one of the studies. See methods for description of weights.

|  | **CFAS I** | | **CFAS II** | |
| --- | --- | --- | --- | --- |
| **Service Counts** | IRR | 95% CI | IRR | 95% CI |
| Any Day to Day Service (count) | 1.2 | 0.3 – 6.0 | 1.1 | 0.3 – 3.6 |
| Any Service in prior four weeks (count) | 1.5 | 0.6 – 4.0 | 0.7 | 0.4 – 1.3 |
| Outpatient in last 3 months (count) | 0.8 | 0.2 – 3.9 | 0.3 | 0.0 – 2.0 |
| Day patient in last year (count) | . | . | . | . |
| Inpatient in last year (count) | . | . | 0.8 | 0.1 – 5.1 |
| **Individual Services** | OR | 95% CI | OR | 95% CI |
| **Day to day services** |  |  |  |  |
| Home help | . | . | . | . |
| Care Worker | . | . | 2.7 | 0.2 – 33.3 |
| Warden | . | . | . | . |
| Paid Help | . | . | 0.5 | 0.1 – 5.2 |
| Any professional day to day service | 1.4 | 0.1 – 21.1 | 1.1 | 0.2 – 6.6 |
| Unpaid Care | 0.5 | 0.0 – 9.2 | 2.6 | 0.3 – 26.3 |
| **Services in prior four weeks** |  |  |  |  |
| Home help/Home care assistant | . | . | 3.8 | 0.5 – 28.3 |
| Any nursing service | 1.0 | 0.1 – 19.6 | 0.6 | 0.1 – 4.0 |
| Chiropodist | 0.7 | 0.0 – 12.7 | 0.5 | 0.1 – 3.0 |
| Physiotherapy | . | . | 0.6 | 0.1 – 6.1 |
| Day Centre | . | . | . | . |
| GP | . | . | 1.0 | 0.2 – 5.3 |
| Any Service | 0.6 | 0.0 – 9.5 | 1.2 | 0.1 – 12.1 |
| **Hospital Services** |  |  |  |  |
| Casualty/outpatient | 1.5 | 0.1 – 29.8 | 0.3 | 0.0 – 3.2 |
| Day patient | . | . | . | . |
| Inpatient | . | . | 0.6 | 0.1 – 6.2 |

**Hypertension**

**Table S18:** Risk ratio and 95% confidence interval comparing hypertension and dementia to hypertension by itself, adjusted for deprivation. Any services which did not have the numbers to calculate risk in either CFAS I or CFAS II were omitted from the table, ‘.’ Represents unable to model risk in one of the studies. See methods for description of weights.

|  | **CFAS I** | | **CFAS II** | |
| --- | --- | --- | --- | --- |
| **Service Counts** | IRR | 95% CI | IRR | 95% CI |
| Any Day to Day Service (count) | 2.1 | 1.2 – 3.5 | 1.7 | 1.2 – 2.4 |
| Any Service in prior four weeks (count) | 1.3 | 1.0 – 1.8 | 1.2 | 0.9 – 1.4 |
| Outpatient in last 3 months (count) | 0.5 | 0.2 – 1.2 | 0.5 | 0.4 – 0.8 |
| Day patient in last year (count) | . | . | 0.5 | 0.2 – 1.5 |
| Inpatient in last year (count) | 2.5 | 1.0 – 6.2 | 1.1 | 0.7 – 1.8 |
| **Individual Services** | OR | 95% CI | OR | 95% CI |
| **Day to day services** |  |  |  |  |
| Home help | 2.0 | 0.4 – 8.6 | 0.8 | 0.2 – 3.4 |
| Care Worker | 5.4 | 2.2 – 12.9 | 9.0 | 5.0 – 16.3 |
| Meals on wheels | 9.4 | 1.7 – 50.8 | 2.7 | 0.3 – 22.3 |
| Community nurse | . | . | 2.3 | 0.3 – 18.5 |
| Warden | . | . | 2.5 | 0.6 – 11.6 |
| Paid Help | 0.8 | 0.3 – 2.6 | 0.7 | 0.3 – 1.4 |
| Other professional services | . | . | 0.8 | 0.1 – 6.2 |
| Any professional day to day service | 2.9 | 1.4 – 6.0 | 1.6 | 1.0 – 2.6 |
| Unpaid Care | 2.6 | 1.3 – 5.5 | 3.6 | 2.2 – 5.9 |
| **Services in prior four weeks** |  |  |  |  |
| Home help/Home care assistant | 2.2 | 0.7 – 6.8 | 6.0 | 3.4 – 10.5 |
| Any nursing service | 1.4 | 0.7 – 3.1 | 1.7 | 1.0 – 2.8 |
| Chiropodist | 0.7 | 0.3 – 1.7 | 1.1 | 0.7 – 1.7 |
| Meals on Wheels | 4.6 | 1.6 – 13.1 | 1.3 | 0.3 – 5.6 |
| Physiotherapist | 3.6 | 0.5 – 27.4 | 0.9 | 0.3 – 2.5 |
| Occupational therapist | . | . | 2.6 | 0.8 – 9.0 |
| Speech therapist | . | . | 1.7 | 0.2 – 14.9 |
| Social worker | 1.4 | 0.2 – 11.3 | 1.9 | 0.2 – 16.6 |
| Day Centre | 3.1 | 1.2 – 8.3 | 7.9 | 4.1 – 15.1 |
| GP | 0.9 | 0.4 – 1.8 | 1.2 | 0.8 – 1.8 |
| Any Service | 1.4 | 0.7 – 3.0 | 1.0 | 0.6 – 1.5 |
| **Hospital Services** |  |  |  |  |
| Casualty/outpatient | 0.8 | 0.3 – 2.2 | 0.7 | 0.5 – 1.2 |
| Day patient | . | . | 0.5 | 0.2 – 1.2 |
| Inpatient | 2.2 | 0.9 – 5.3 | 1.0 | 0.6 – 1.6 |

**Anaemia**

**Table S19:** Risk ratio and 95% confidence interval comparing anaemia and dementia to anaemia by itself, adjusted for deprivation. Any services which did not have the numbers to calculate risk in either CFAS I or CFAS II were omitted from the table, ‘.’ Represents unable to model risk in one of the studies. See methods for description of weights.

|  | **CFAS I** | | **CFAS II** | |
| --- | --- | --- | --- | --- |
| **Service Counts** | IRR | 95% CI | IRR | 95% CI |
| Any Day to Day Service (count) | 2.2 | 1.2 – 4.4 | 1.3 | 0.5 – 3.3 |
| Any Service in prior four weeks (count) | 1.4 | 0.9 – 2.2 | 0.9 | 0.5 – 1.8 |
| Outpatient in last 3 months (count) | . | . | 0.3 | 0.1 – 0.8 |
| Day patient in last year (count) | . | . | 0.7 | 0.4 – 1.3 |
| Inpatient in last year (count) | 0.4 | 0.1 – 1.8 | 1.3 | 0.3 – 4.9 |
| **Individual Services** | OR | 95% CI | OR | 95% CI |
| **Day to day services** |  |  |  |  |
| Home help | . | . | . | . |
| Care Worker | 3.7 | 0.8 – 17.3 | 10.0 | 2.6 – 38.9 |
| Warden | . | . | . | . |
| Paid Help | . | . | . | . |
| Any professional day to day service | 8.3 | 1.5 – 46.4 | 1.5 | 0.5 – 4.6 |
| Unpaid Care | 5.7 | 0.6 – 50.1 | 2.0 | 0.6 – 6.8 |
| **Services in prior four weeks** |  |  |  |  |
| Home help/Home care assistant | 7.5 | 1.6 – 34.4 | 3.0 | 0.7 – 13.4 |
| Any nursing service | 0.5 | 0.1 – 2.5 | 3.4 | 1.0 – 11.6 |
| Chiropodist | 2.5 | 0.6 – 11.0 | 1.6 | 0.5 – 5.3 |
| Meals on wheels | 1.1 | 0.1 – 10.3 | . | . |
| Physiotherapist | . | . | 1.2 | 0.1 – 10.5 |
| Occupational therapist | . | . | 3.9 | 0.3 – 44.4 |
| Social worker | 1.9 | 0.2 – 21.7 | . | . |
| Day Centre | 2.4 | 0.3 – 16.4 | 4.7 | 0.7 – 31.6 |
| Day hospital | . | . | 2.5 | 0.3 – 24.2 |
| GP | 0.8 | 0.2 – 3.4 | 0.4 | 0.1 – 1.5 |
| Any Service | 2.3 | 0.3 – 20.9 | 0.6 | 0.2 – 1.8 |
| **Hospital Services** |  |  |  |  |
| Casualty/outpatient | 0.2 | 0.0 – 1.5 | 0.5 | 0.1 – 2.0 |
| Day patient | 0.6 | 0.1 – 5.2 | 1.7 | 0.4 – 7.0 |
| Inpatient | 0.8 | 0.1 – 4.3 | 0.6 | 0.1 – 2.8 |

**Breathing difficulties**

**Table S20:** Risk ratio and 95% confidence interval comparing breathing difficulties and dementia to breathing difficulties by itself, adjusted for deprivation. Any services which did not have the numbers to calculate risk in either CFAS I or CFAS II were omitted from the table, ‘.’ Represents unable to model risk in one of the studies. See methods for description of weights.

|  | **CFAS I** | | **CFAS II** | |
| --- | --- | --- | --- | --- |
| **Service Counts** | IRR | 95% CI | IRR | 95% CI |
| Any Day to Day Service (count) | 1.4 | 0.9 – 2.3 | 1.5 | 0.8 – 2.8 |
| Any Service in prior four weeks (count) | 1.3 | 0.9 – 1.8 | 1.1 | 0.8 – 1.6 |
| Outpatient in last 3 months (count) | 0.8 | 0.3 – 1.9 | 0.5 | 0.3 – 1.1 |
| Day patient in last year (count) | . | . | 1.1 | 0.3 – 4.9 |
| Inpatient in last year (count) | 2.3 | 1.1 – 4.8 | 2.0 | 1.0 – 3.8 |
| **Individual Services** | OR | 95% CI | OR | 95% CI |
| **Day to day services** |  |  |  |  |
| Home help | . | . | 1.7 | 0.2 – 14.2 |
| Care Worker | 5.8 | 2.0 – 16.4 | 4.7 | 1.6 – 14.0 |
| Warden | . | . | 2.3 | 0.3 – 18.4 |
| Paid Help | 0.8 | 0.2 – 3.0 | 1.3 | 0.5 – 3.3 |
| Any professional day to day service | 2.2 | 0.9 – 5.3 | 1.6 | 0.7 – 3.5 |
| Unpaid Care | 1.2 | 0.5 – 2.9 | 2.1 | 1.0 – 4.5 |
| **Services in prior four weeks** |  |  |  |  |
| Home help/Home care assistant | 3.9 | 1.3 – 11.4 | 3.8 | 1.5 – 9.8 |
| Any nursing service | 2.2 | 0.8 – 6.0 | 2.9 | 1.3 – 6.5 |
| Chiropodist | 1.7 | 0.7 – 4.6 | 1.2 | 0.5 – 2.6 |
| Meals on wheels | 5.0 | 1.4 – 17.3 | . | . |
| Physiotherapist | . | . | 1.2 | 0.3 – 5.1 |
| Occupational therapist | . | . | 3.2 | 0.7 – 14.8 |
| Day Centre | 1.4 | 0.3 – 6.6 | 4.2 | 1.2 – 15.4 |
| Day Hospital | 5.6 | 1.0 – 31.6 | . | . |
| GP | 0.7 | 0.3 – 1.9 | 1.1 | 0.5 – 2.4 |
| Any Service | 1.1 | 0.4 – 2.8 | 0.7 | 0.3 – 1.4 |
| **Hospital Services** |  |  |  |  |
| Casualty/outpatient | 0.7 | 0.3 – 2.1 | 0.6 | 0.3 – 1.5 |
| Day patient | . | . | 0.7 | 0.2 – 2.5 |
| Inpatient | 2.7 | 1.0 – 7.0 | 1.6 | 0.7 – 3.6 |

**Transient ischaemic attack**

**Table S21:** Risk ratio and 95% confidence interval comparing transient ischaemic attack and dementia to transient ischaemic attack by itself, adjusted for deprivation. Any services which did not have the numbers to calculate risk in either CFAS I or CFAS II were omitted from the table, ‘.’ Represents unable to model risk in one of the studies. See methods for description of weights.

|  | **CFAS I** | | **CFAS II** | |
| --- | --- | --- | --- | --- |
| **Service Counts** | IRR | 95% CI | IRR | 95% CI |
| Any Day to Day Service (count) | 2.1 | 1.3 – 3.5 | 2.4 | 1.5 – 3.7 |
| Any Service in prior four weeks (count) | 1.2 | 0.8 – 1.8 | 1.3 | 1.0 – 1.8 |
| Outpatient in last 3 months (count) | 0.8 | 0.4 – 1.7 | 0.5 | 0.3 – 0.9 |
| Day patient in last year (count) | . | . | 0.8 | 0.5 – 1.3 |
| Inpatient in last year (count) | 3.4 | 1.6 – 7.1 | 1.5 | 0.9 – 2.7 |
| **Individual Services** | OR | 95% CI | OR | 95% CI |
| **Day to day services** |  |  |  |  |
| Home help | 3.0 | 0.8 – 11.6 | 2.1 | 0.4 – 10.6 |
| Care Worker | 6.1 | 2.4 – 15.5 | 8.9 | 3.9 – 20.4 |
| Community nurse | . | . | 4.8 | 0.5 – 51.4 |
| Warden | . | . | . | . |
| Paid Help | 1.0 | 0.3 – 3.2 | 1.0 | 0.4 – 2.5 |
| Other professional services | . | . | 0.7 | 0.1 – 6.0 |
| Any professional day to day service | 2.7 | 1.2 – 6.0 | 2.7 | 1.4 – 5.0 |
| Unpaid Care | 1.2 | 0.5 – 2.6 | 2.5 | 1.2 – 5.1 |
| **Services in prior four weeks** |  |  |  |  |
| Home help/Home care assistant | 5.2 | 2.1 – 13.2 | 5.3 | 2.4 – 11.3 |
| Any nursing service | 1.0 | 0.4 – 2.6 | 1.6 | 0.8 – 3.5 |
| Chiropodist | 0.8 | 0.3 – 2.0 | 1.1 | 0.6 – 2.2 |
| Meals on Wheels | 3.9 | 1.0 – 14.6 | 1.9 | 0.2 – 18.5 |
| Physiotherapist | 5.4 | 0.7 – 41.5 | 1.1 | 0.4 – 3.3 |
| Occupational therapist | . | . | 4.1 | 0.7 – 25.9 |
| Speech therapist | . | . | 1.8 | 0.2 – 22.1 |
| Social worker | 1.2 | 0.1 – 9.9 | 2.0 | 0.2 – 25.6 |
| Day Centre | 2.5 | 0.8 – 7.5 | 7.8 | 3.0 – 20.2 |
| Day hospital | 6.7 | 1.0 – 43.7 | 1.0 | 0.1 – 8.0 |
| GP | 1.1 | 0.5 – 2.5 | 1.1 | 0.6 – 2.1 |
| Any Service | 0.7 | 0.3 – 1.5 | 1.4 | 0.7 – 2.8 |
| **Hospital Services** |  |  |  |  |
| Casualty/outpatient | 1.4 | 0.5 – 3.8 | 0.5 | 0.3 – 1.2 |
| Day patient | 0.5 | 0.1 – 2.4 | 1.3 | 0.6 – 2.8 |
| Inpatient | 3.3 | 1.3 – 8.5 | 1.5 | 0.7 – 3.0 |

**Heart attack**

**Table S22:** Risk ratio and 95% confidence interval comparing heart attack and dementia to heart attack by itself, adjusted for deprivation. Any services which did not have the numbers to calculate risk in either CFAS I or CFAS II were omitted from the table, ‘.’ Represents unable to model risk in one of the studies. See methods for description of weights.

|  | **CFAS I** | | **CFAS II** | |
| --- | --- | --- | --- | --- |
| **Service Counts** | IRR | 95% CI | IRR | 95% CI |
| Any Day to Day Service (count) | 1.9 | 0.7 – 4.8 | 1.8 | 1.0 – 3.1 |
| Any Service in prior four weeks (count) | 1.2 | 0.6 – 2.2 | 1.2 | 0.9 – 1.7 |
| Outpatient in last 3 months (count) | 1.0 | 0.4 – 2.7 | 0.3 | 0.1 – 0.6 |
| Day patient in last year (count) | . | . | 1.4 | 0.6 – 3.3 |
| Inpatient in last year (count) | 2.7 | 0.9 – 7.8 | 1.1 | 0.6 – 2.0 |
| **Individual Services** | OR | 95% CI | OR | 95% CI |
| **Day to day services** |  |  |  |  |
| Home help | . | . | 2.5 | 0.7 – 9.5 |
| Care Worker | 7.3 | 1.6 – 33.5 | 13.3 | 5.5 – 32.2 |
| Warden | . | . | 3.2 | 0.3 – 32.4 |
| Paid Help | 0.4 | 0.0 – 3.2 | 0.5 | 0.2 – 1.8 |
| Any professional day to day service | 2.0 | 0.5 – 7.7 | 1.7 | 0.8 – 3.5 |
| Unpaid Care | 0.6 | 0.2 – 2.3 | 2.9 | 1.3 – 6.5 |
| **Services in prior four weeks** |  |  |  |  |
| Home help/Home care assistant | 4.3 | 0.9 – 20.9 | 6.2 | 2.7 – 13.9 |
| Any nursing service | 0.3 | 0.0 – 2.4 | 1.0 | 0.4 – 2.3 |
| Chiropodist | 1.3 | 0.3 – 5.5 | 1.1 | 0.5 – 2.2 |
| Meals on wheels | 7.2 | 1.2 – 42.7 | . | . |
| Physiotherapist | . | . | 0.4 | 0.1 – 3.2 |
| Day Centre | . | . | 6.8 | 2.1 – 22.1 |
| Day hospital | . | . | 2.9 | 0.6 – 14.3 |
| GP | 0.3 | 0.1 – 1.3 | 1.3 | 0.6 – 2.5 |
| Any Service | 1.0 | 0.3 – 3.6 | 1.3 | 0.5 – 3.0 |
| **Hospital Services** |  |  |  |  |
| Casualty/outpatient | 1.7 | 0.3 – 8.6 | 0.4 | 0.2 – 1.0 |
| Day patient | . | . | 0.5 | 0.2 – 1.3 |
| Inpatient | 2.1 | 0.4 – 9.6 | 0.9 | 0.4 – 2.0 |

**Hearing difficulties**

**Table S23:** Risk ratio and 95% confidence interval comparing hearing difficulties and dementia to hearing difficulties by itself, adjusted for deprivation. Any services which did not have the numbers to calculate risk in either CFAS I or CFAS II were omitted from the table, ‘.’ Represents unable to model risk in one of the studies. See methods for description of weights.

|  | **CFAS I** | | **CFAS II** | |
| --- | --- | --- | --- | --- |
| **Service Counts** | IRR | 95% CI | IRR | 95% CI |
| Any Day to Day Service (count) | 1.2 | 0.6 – 2.2 | 1.5 | 1.1 – 2.1 |
| Any Service in prior four weeks (count) | 0.9 | 0.5 – 1.6 | 1.1 | 0.9 – 1.4 |
| Outpatient in last 3 months (count) | 0.4 | 0.1 – 1.6 | 0.6 | 0.4 – 0.9 |
| Day patient in last year (count) | . | . | 0.6 | 0.3 – 1.3 |
| Inpatient in last year (count) | 1.3 | 0.5 – 3.4 | 1.0 | 0.6 – 1.6 |
| **Individual Services** | OR | 95% CI | OR | 95% CI |
| **Day to day services** |  |  |  |  |
| Home help | 2.6 | 0.3 – 20.6 | 1.6 | 0.6 – 4.2 |
| Care Worker | 2.8 | 0.8 – 9.1 | 6.2 | 3.4 – 11.4 |
| Meals on wheels | . | . | 1.7 | 0.2 – 14.8 |
| Community worker | . | . | 3.7 | 0.4 – 31.2 |
| Community nurse | . | . | 1.2 | 0.1 – 10.3 |
| Warden | . | . | 2.1 | 0.6 – 7.7 |
| Paid Help | 0.9 | 0.2 – 4.4 | 0.5 | 0.2 – 1.1 |
| Any professional day to day service | 1.6 | 0.6 – 4.3 | 1.8 | 1.1 – 2.9 |
| Unpaid Care | 1.4 | 0.5 – 4.0 | 2.4 | 1.4 – 3.8 |
| **Services in prior four weeks** |  |  |  |  |
| Home help/Home care assistant | 2.1 | 0.6 – 7.0 | 5.3 | 3.0 – 9.5 |
| Any nursing service | 0.5 | 0.1 – 2.1 | 1.3 | 0.8 – 2.3 |
| Chiropodist | 1.2 | 0.4 – 3.6 | 1.1 | 0.6 – 1.8 |
| Meals on Wheels | 1.4 | 0.3 – 6.7 | 0.9 | 0.2 – 4.0 |
| Physiotherapist | . | . | 0.4 | 0.1 – 1.5 |
| Occupational therapist | . | . | 0.8 | 0.1 – 6.7 |
| Day Centre | 1.5 | 0.3 – 7.4 | 6.8 | 2.9 – 15.9 |
| Day hospital | . | . | 5.4 | 1.5 – 19.0 |
| GP | 1.0 | 0.3 – 2.9 | 0.8 | 0.5 – 1.3 |
| Any Service | 0.5 | 0.2 – 1.4 | 0.9 | 0.5 – 1.4 |
| **Hospital Services** |  |  |  |  |
| Casualty/outpatient | 0.3 | 0.1 – 1.6 | 0.8 | 0.5 – 1.3 |
| Day patient | 0.6 | 0.1 – 4.4 | 0.7 | 0.3 – 1.4 |
| Inpatient | 1.6 | 0.5 – 4.9 | 0.9 | 0.5 – 1.5 |

**Any target comorbidity**

**Table S24:** Risk ratio and 95% confidence interval comparing any target comorbidity and dementia to any target comorbidity by itself, adjusted for deprivation. Any services which did not have the numbers to calculate risk in either CFAS I or CFAS II were omitted from the table, ‘.’ Represents unable to model risk in one of the studies. See methods for description of weights.

|  | **CFAS I** | | **CFAS II** | |
| --- | --- | --- | --- | --- |
| **Service Counts** | IRR | 95% CI | IRR | 95% CI |
| Any Day to Day Service (count) | 2.1 | 1.4 – 3.3 | 1.4 | 1.0 – 2.0 |
| Any Service in prior four weeks (count) | 1.1 | 0.8 – 1.6 | 1.5 | 1.1 – 2.0 |
| Outpatient in last 3 months (count) | 0.5 | 0.2 – 1.2 | 0.5 | 0.3 – 0.7 |
| Day patient in last year (count) | . | . | 1.0 | 0.4 – 2.4 |
| Inpatient in last year (count) | 2.8 | 1.3 – 5.9 | 1.5 | 1.0 – 2.3 |
| **Individual Services** | OR | 95% CI | OR | 95% CI |
| **Day to day services** |  |  |  |  |
| Home help | 2.2 | 0.5 – 10.4 | 1.8 | 0.7 – 4.8 |
| Care worker | 4.9 | 2.1 – 11.4 | 6.7 | 3.6 – 12.4 |
| Community nurse | . | . | 5.9 | 1.2 – 29.9 |
| Warden | . | . | 0.7 | 0.1 – 5.0 |
| Paid help | 1.1 | 0.4 – 3.2 | 0.3 | 0.1 – 0.8 |
| Other day to day services | . | . | 0.7 | 0.1 – 5.5 |
| Any service | 2.9 | 1.4 – 5.8 | 1.9 | 1.2 – 3.1 |
| Unpaid care | 1.0 | 0.5 – 2.0 | 2.9 | 1.8 – 4.8 |
| **Services in prior four weeks** |  |  |  |  |
| Home help/home care assistant | 3.4 | 1.4 – 8.6 | 4.7 | 2.6 – 8.7 |
| Any nursing Services | 1.1 | 0.5 – 2.4 | 2.2 | 1.3 – 3.8 |
| Chiropodist | 0.6 | 0.3 – 1.5 | 1.7 | 1.0 – 2.8 |
| Meals on wheels | 5.9 | 2.2 – 15.7 | 0.5 | 0.1 – 3.7 |
| Physiotherapist | 5.7 | 0.7 – 46.6 | 0.6 | 0.2 – 2.1 |
| Occupational Therapist | . | . | 2.1 | 0.6 – 7.5 |
| Day Centre | 2.5 | 0.9 – 7.0 | 7.5 | 3.6 – 15.5 |
| Day Hospital | 9.3 | 1.8 – 49.6 | 5.9 | 1.6 – 21.9 |
| GP | 0.9 | 0.4 – 1.9 | 1.1 | 0.7 – 1.7 |
| Any service | 0.6 | 0.3 – 1.2 | 1.3 | 0.8 – 2.2 |
| **Hospital Services** |  |  |  |  |
| Casualty/Outpatient | 0.8 | 0.3 – 2.1 | 0.6 | 0.3 – 1.1 |
| Day patient | 0.2 | 0.0 – 1.5 | 0.6 | 0.3 – 1.3 |
| Inpatient | 3.1 | 1.3 – 7.3 | 1.6 | 0.9 – 2.7 |
